# Plasmonic Multichannel Refractive Index Sensor Based on Subwavelength Tangent-Ring Metal–Insulator–Metal Waveguide

**DOI:** 10.3390/s18051348

**Published:** 2018-04-26

**Authors:** Zicong Guo, Kunhua Wen, Qinyang Hu, Wenhui Lai, Jiyan Lin, Yihong Fang

**Affiliations:** School of Physics and Optoelectronic Engineering, Guangdong University of Technology, Guangzhou 510006, Guangdong, China; guoguo19960423@163.com (Z.G.); keyonwho@163.com (Q.H.); Wenhuibn@163.com (W.L.); linjiyan2819@163.com (J.L.); fongyathong@yeah.net (Y.F.)

**Keywords:** refractive index sensor, sub-wavelength MIM waveguide, Fano resonance

## Abstract

In this paper, a multichannel refractive index sensor based on a subwavelength metal–insulator–metal (MIM) waveguide coupled with tangent-ring resonators is proposed. When two tangent-ring resonators were placed above the MIM waveguide, Fano resonance with asymmetrical line shape appeared in the transmission spectrum due to the interference between the light–dark resonant modes. The sensitivity and figure of merit were as high as 880 nm/RIU and 964, respectively. Through adding more tangent-ring resonators, multiple Fano resonances with ultrasharp peaks/dips were achieved in the wavelength range of 800–2000 nm. Besides, negative group delays were also observed in the Fano resonant dips. Two-dimensional finite-difference time-domain (FDTD) method was used to simulate and analyze the performances of the proposed structures. These kinds of multiring structures can find important applications in the on-chip optical sensing and optical communication areas.

## 1. Introduction

Subwavelength metal–insulator–metal (MIM) waveguides based on surface plasmon polariton (SPP), which is one of the promising methods to control the light transmission at nanoscale, have vigorously pushed forward the development of integrated photonics circuits [1]. Various nanoscale optical devices based on MIM waveguides have been proposed and demonstrated, such filters, splitters, sensors, and so on [2,3,4,5,6,7,8]. In particular, Fano resonances [9,10,11,12] which were previously demonstrated in the atom’s system and are caused by the coherent coupling and interference between a discrete state and a continuous state—have also been investigated in MIM waveguides. Due to the advantages of asymmetrical ultrasharp line shapes and high figure of merit (FOM), Fano resonances are quite preferred in the optical sensing, laser, and optical signal detection areas [13,14,15,16,17,18,19,20,21]. In the MIM waveguide structures, Fano resonances can be generated by the interference between the dark mode (corresponding to the discrete excited state) and the radiative bright mode (corresponding to the continuum state). For example, sharp and asymmetric Fano-line spectra are found in the MIM waveguides with dual side-coupled slot cavities or dual parallel grooves [22,23,24]. In the former researches, single Fano resonance was firstly investigated to obtain the high performances of FOM and refractive-index sensitivity, and then dual Fano resonance was investigated in the MIM structures to improve the on-chip integration [25,26]. In this case, more Fano peaks/dips are preferred in the single MIM structure to satisfy the development of a high-integrated photonic circuit.

In this study, multiple Fano resonances were achieved in a MIM waveguide structure composed of several tangent-ring resonators. Since the radius of each resonant ring was different, distinct resonant modes could generated respectively. According to the interferences between the bright modes and the dark modes, multiple Fano peaks with asymmetric line shapes were achieved. The results show that the number of Fano peaks depends largely on the number of coupling loops around the rings. Although this proposed tangent-ring resonator is a little more complicated than the stubs structures, which support single Fano resonance [27,28], more Fano channels can be obtained with the proposed method. This kind of structure can be used as a multichannel on-chip sensor, and it also meets the development of integrated photonics. Finite-difference time-domain (FDTD) method was employed to investigate the performance of the proposed structures.

## 2. Structure and Discussion

**Figure 1:**
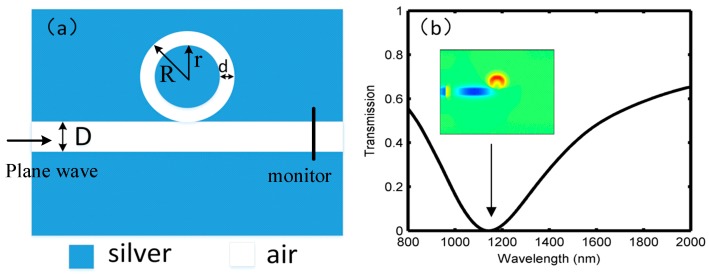
(**a**) Single ring resonator structure; and (**b**) transmission spectrum.

The single ring resonator, which is shown in Figure 1a, is a conventional Fabry–Perot (FP) resonator located at one side of the MIM waveguide. The widths of the MIM waveguide and the ring resonator are denoted by *D* and *d*, respectively. The inner and outer radii of the ring are defined as *r* and *R*, respectively. Thus, the resonant conditions can be approximately given by

(1) λm=2Re(neff)Leffm,m=1, 2, 3, ⋯

where $L_{eff}=\pi \left(r+R\right)$ is the effective resonance length, Re(*n_eff_*) is the real part of the effective refractive index, and *n_eff_* obtained from the dispersion equations [29].

During the FDTD simulations, the commercial tool “FDTD Solutions” was used. The input light was defined as a plane light wave, and a monitor was set at the output MIM waveguide, as indicated in Figure 1a. The perfect matching layers (PMLs) were set around the structure to absorb the escaping electromagnetic field energy, and the number of layers in x- and y-directions were defined as 64. The following parameters were unchanged throughout the study: the width of the MIM waveguide *D* = 50 nm, the thickness of the ring resonator *d* = 20 nm, the inner and outer diameters *r* = 40 nm and *R* = 60 nm, respectively. The ring resonator was connected to the MIM waveguide directly (i.e., the spacing distance was 0 nm). A uniform set of perfect matching layers, which were used as the absorption boundary condition, was employed in the structure. The metal and dielectric materials were firstly defined as silver and air, respectively, and the permittivity factors were obtained from the experimental data [30]. The transmission spectrum for the ring resonator structure is shown in Figure 1b, which produces a symmetrical Lorentzian line type. This kind of FP resonator can perform as a band-stop filter, whose forbidden band is generated at 1140 nm. Observed from the corresponding magnetic field at the center wavelength was a strong magnetic field in the upper half of the ring resonator, and almost none of the SPP energy was distributed in the output side of the MIM waveguide.

**Figure 2:**
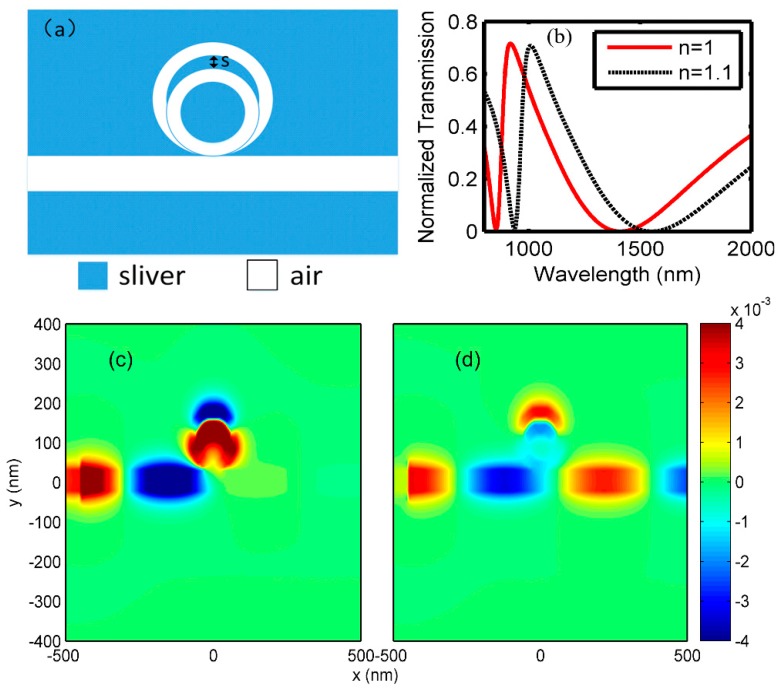
(**a**) Double-ring resonator structure; (**b**) transmission spectrum; (**c**) magnetic field at dip λ=854 nm trough; and (**d**) magnetic field at peak λ=920 nm.

In Figure 2a, a ring with the inner and outer radii of 60 nm and 80 nm, respectively, was added into the former resonator of Figure 1a. The two rings were aligned with the normal line, while their bottoms were tangent. Since the two rings were tangent, “s” parameter was calculated as 20 nm, according the radii of the rings, and it remained unchanged between the adjacent two rings throughout the study. According to the FP resonant conditions, different resonant modes were excited in the two rings, respectively. Specifically, a bright mode with broad bandwidth and a dark mode with narrow bandwidth will arise in the small ring and large ring, respectively. Due to the interference between the bright and dark modes, Fano resonance with an asymmetric line shape was generated. The simulation spectrum, plotted with the red-dotted line, is shown in Figure 2b, which illustrates that a transmission peak with a transmittance of ~0.7 was generated at 920 nm. A steep dip, which had the lowest transmittance of ~0 at 854 nm, occurred at the left side of the transmission peak. Contrarily, the transmission at the right side of the peak changed slowly, and the trough arose at 1400 nm. When the insulator was changed to the one with the refractive index of 1.1, the transmission peak and dip shifted to 1008 nm and 936 nm, respectively. Subsequently, a high sensitivity *S* of 880 nm/RIU for the refractive index was achieved based on Equation (2). This is a promising characteristic for the on-chip sensor.

(2) $$S=\frac{d\lambda }{dn\left(\lambda \right)}$$

Additionally, figure of merit (FOM) was also significantly factored to evaluate the performances of the sensor, and it can be expressed as [26]:

(3) $$\mathrm{FOM}=\max\left(\left|\frac{dT\left(\lambda \right)/dn\left(\lambda \right)}{T\left(\lambda \right)}\right|\right)$$

where T(λ) is the transmission, and dT(λ)/dn(λ) is the transmittance change at fixed wavelength induced by a refractive index change. According to Equation (3), it can be concluded that an ultralow transmittance followed by a sharp increase induced by the changes in index is preferred for obtaining a high FOM. In this proposed structure, a high FOM of 964 was achieved at the dip.

Figure 2c, which shows the magnetic field of the dip at 854 nm, indicates that a strong magnetic field is distributed at the input MIM waveguide and essentially none of magnetic field is presented at the output waveguide. In addition, strong magnetic fields occur at both side-coupled rings, leading to a strong resonance and a forbidden band. Figure 2d is the magnetic field distribution corresponding to the Fano resonance peak at 920 nm. The output waveguide has a strong magnetic field, which is in accordance with the transmission spectrum. Comparing to Figure 2c, Figure 2d shows that weaker magnetic fields are distributed in both rings, resulting a transmission peak. 

**Figure 3:**
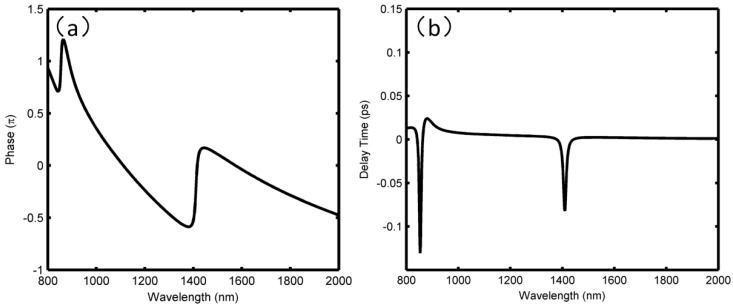
(**a**) Phase responses; and (**b**) delay time for the double ring resonator structure, respectively.

The phase responses and the group delays are studied in Figure 3. Particularly, the phase changes between 800 nm and 2000 nm are plotted in Figure 3a, which shows the phase is shifted from 0.75π to 1.25π at the wavelength ranging from 854 to 920 nm, while at the wavelength from 1400 to 1450 nm, the phase jumps from −0.4π to 0.1π. This indicates that the phase continuity will be broken within the transmission dip, but the phase changes linearly in other wavelengths. According to the relationship between the group delay τ and the phase θ, the delay time satisfies the condition: *τ*(λ) = −λ^2^*dθ*/2*πcdλ*, respectively. In view of the phase responses in Figure 3a, it can be concluded that abnormal dispersion will be achieved at the Fano dip. From Figure 3b, we can see that ~−0.13 ps and ~−0.08 ps group delays are obtained at the two dips, respectively.

**Figure 4:**
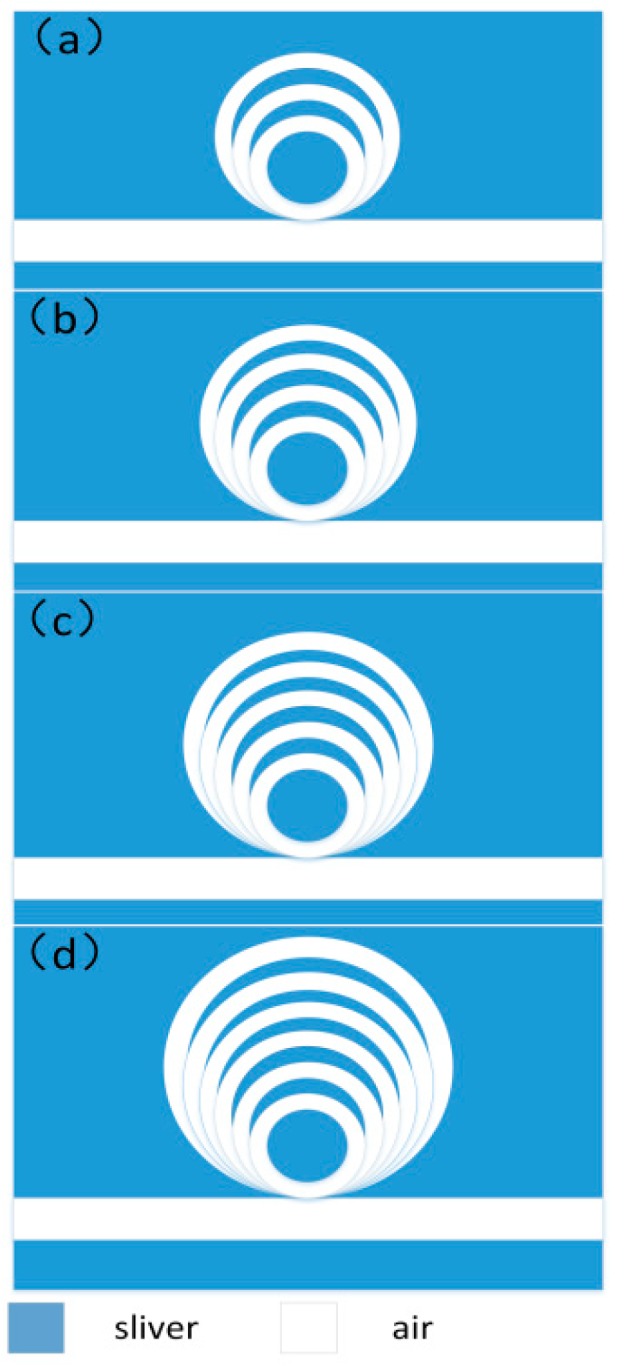
Multiring resonator structure. (**a**) Three rings; (**b**) four rings; (**c**) five rings and (**d**) six rings.

**Figure 5:**
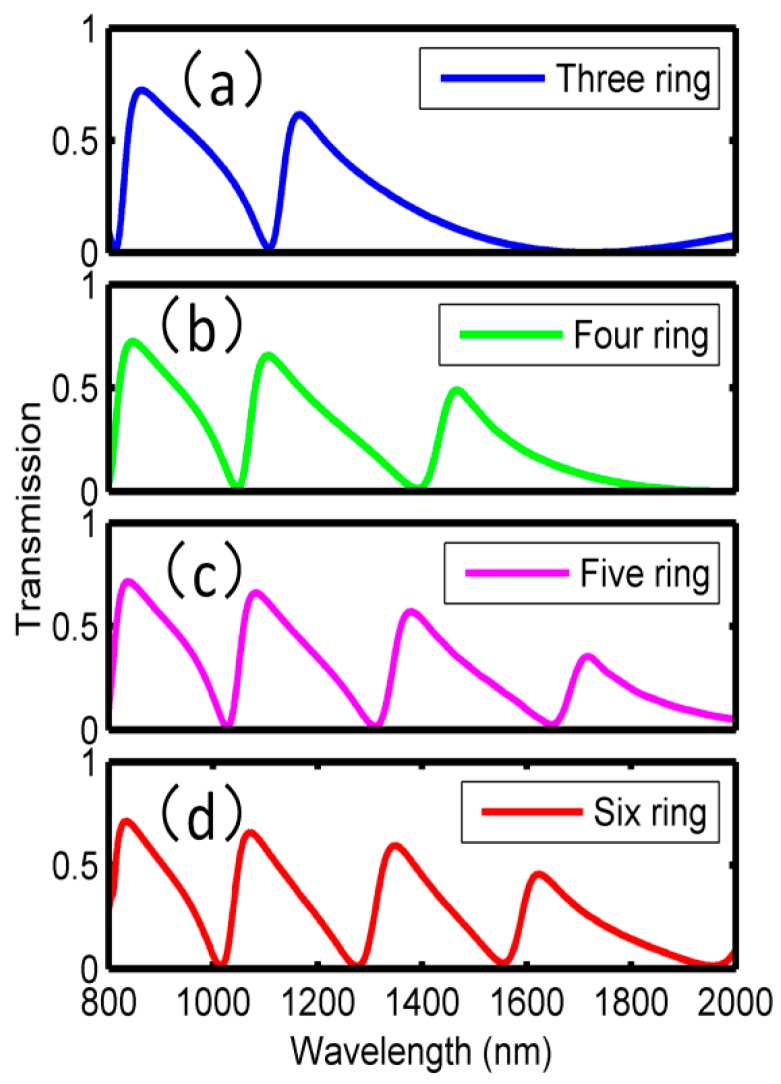
Multiring resonator transmission spectrum.

Furthermore, more bottom-tangent-rings were added on the basis of Figure 2a to obtain more Fano resonant peaks, as shown in Figure 4a–d, where three-, four-, five-, and six-ring resonators were placed above the MIM waveguide, respectively. The outer radii for the newly added rings were increased with a step of 20 nm (i.e., 100 nm, 120 nm, 140 nm and 160 nm, respectively), while their widths were the same, at 20 nm. Usually, single Fano resonance requires a bright mode and a dark mode that interact with each other and exactly two different tangent rings can support a bright mode and a dark mode, respectively. In this case, single Fano resonance was achieved in this dual-tangent-ring resonator, which was investigated as shown in Figure 2. After adding a new ring, one more Fano resonance could be obtained. Subsequently, more Fano resonances can be seen in Figure 4, and the corresponding transmission spectra are shown in Figure 5. Three-, four-, five-, and six-ring structures correspond to two, three, four, and five Fano peaks with asymmetrical transmission line shapes, respectively. Figure 5a–c, showing transmission spectra in the wavelength range of 800–2000 nm, agree well with the analysis above. This phenomenon seems not applicable to Figure 5d in this wavelength range. Actually, when the wavelength is expanded to 800–2600 nm, five Fano peaks can be found. This demonstrates that we can manipulate the Fano resonances through designing the ring resonators, and then more sensing channels can be achieved in this proposed on-chip MIM waveguide structure.

**Figure 6:**
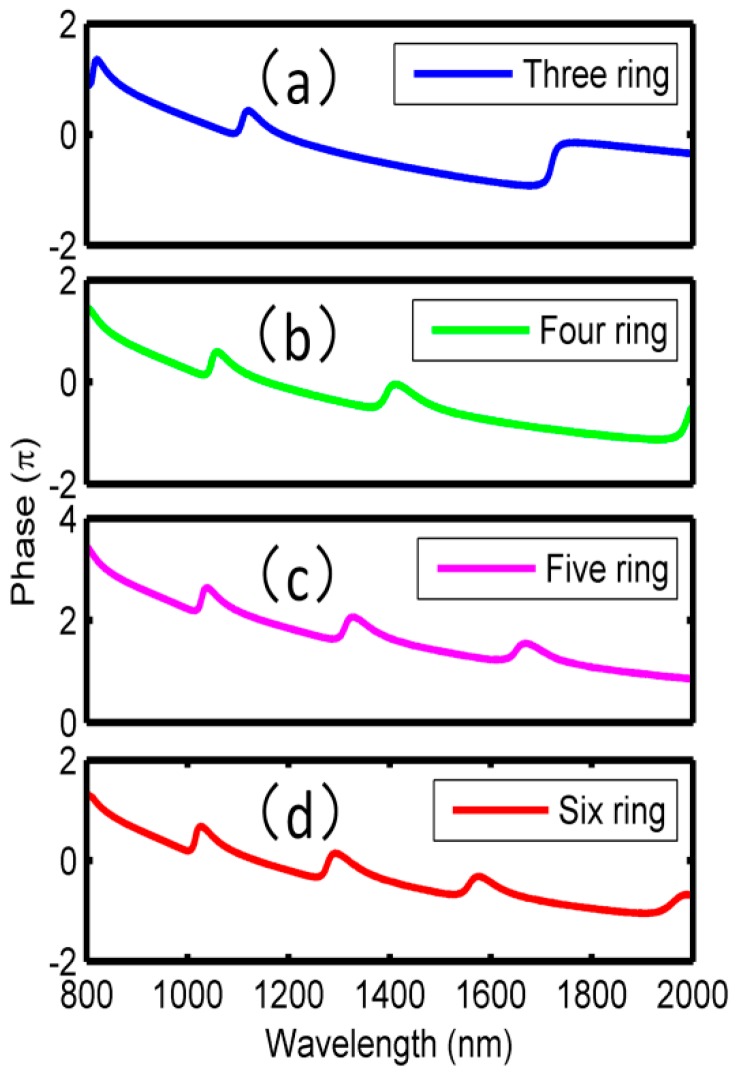
Phase responses of multiring resonators. (**a**) Three rings; (**b**) four rings; (**c**) five rings; and (**d**) six rings.

**Figure 7:**
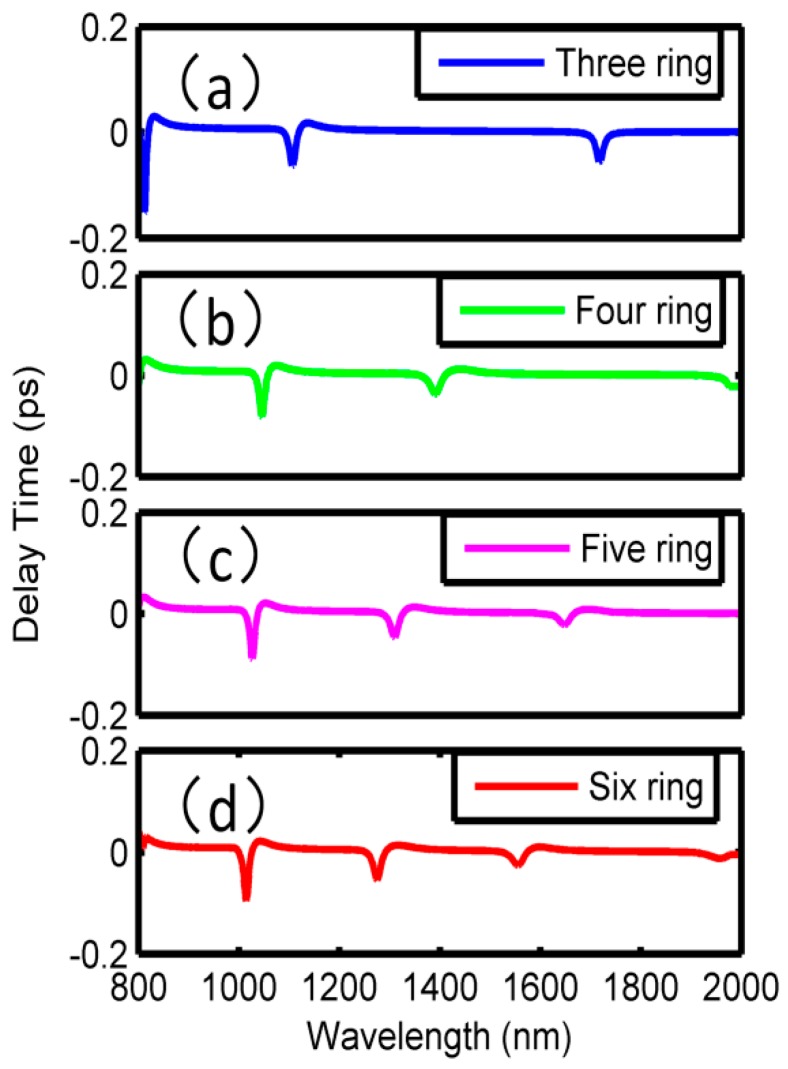
Group delays of multiring resonators.

Moreover, the phase responses and the group delays corresponding to the Fano resonances in Figure 5 are plotted in Figure 6 and Figure 7, respectively. In Figure 6, it can be seen that phase shifts will arise at all the Fano dips, and therefore, obvious negative group delays are observed within the wavelength ranges of the dips in Figure 7. Taking Figure 6a and Figure 7a as an example, the phase changes occurred at 810 nm, 1100 nm and 1700 nm, respectively, corresponding to the Fano resonance dips in Figure 5a. In wavelengths ranging from 810 to 860 nm, 1100 to 1160 nm, and 1700 to 1750 nm, the phases changed from 1.00π to 1.50π, 0 to 0.50π, and −0.80π to 0, respectively. In the other wavelength ranges, the phases changed linearly. Subsequently, the group delays could be calculated, and the maximum negative delays were −0.15 ps, −0.05 ps, and −0.04 ps at 810 nm, 1100 nm and 1700 nm, respectively. More detailed results were shown in Table 1. It is suggested that, in additional to the sensing application, one can also use the proposed structure in the fast light communication area. 

**Figure 8:**
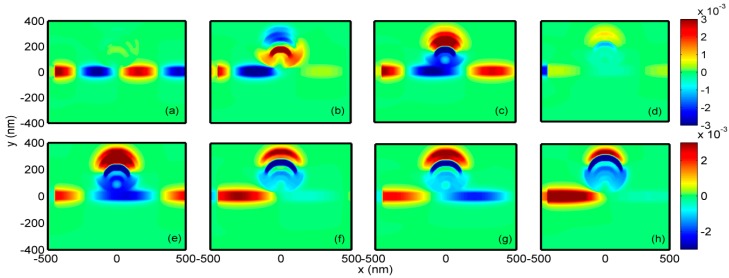
Magnetic field distributions for the six-ring resonator (**a**) peak at 835 nm, (**b**) dip at 1015 nm, (**c**) peak at 1070 nm, (**d**) dip at 1275 nm, (**e**) peak at 1348 nm, (**f**) dip at 1556 nm, (**g**) peak at 1623 nm and (**h**) dip at 1960 nm.

To get insight into the details of the mode interactions that lead to Fano resonances, Figure 8 shows the magnetic field distributions of the six-ring resonator. At the dip wavelengths, the magnetic fields are almost zero at the bottoms of the rings, as shown in Figure 8b,d,f,h, respectively. In the output MIM waveguide, there is also almost no SPP energy. Contrary distribution details can be observed in Figure 8a,c,e,g, which illustrate the magnetic fields at the peak wavelengths. Besides, strong interferences between the modes are also seen inside the rings, leading to multiple Fano resonances. 

## 3. Conclusions

The transmission characteristics of MIM waveguide based on SPPs in multi-tangent-ring resonators were studied. Fano resonances have been achieved according to the interactions between the dark modes and bright modes. High sensitivity of 880 nm/RIU and FOM of 964 were achieved. It has been demonstrated that the Fano resonant channels can be effectively manipulated by adding tangent rings. In addition, negative group delays were observed in the Fano dips, and one can use the proposed structure in the fast-light area. The results were investigated by FDTD simulations, and it is believed that the proposed structure can find wide application in the on-chip optical sensing areas.

## Figures and Tables

**Table 1:** The phase shifts and the group delays corresponding to Figure 6 and Figure 7.

	Number	Wavelength Range		
Phase shifts and group delays	(a)	810 nm–860 nm	1100 nm–1160 nm	1700 nm–1750 nm
		0.5π, −0.15 ps	0.5π, −0.05 ps	0.8π, −0.04 ps
	(b)	1050 nm–1100 nm		1400 nm–1470 nm
		0.5π, −0.08 ps		0.4π, −0.02 ps
	(c)	1030 nm–1080 nm	1310 nm–1380 nm	1650 nm–1720 nm
		0.5π, −0.10 ps	0.1π, −0.05 ps	0.3π, −0.02 ps
	(d)	1015 nm–1070 nm	1280 nm–1350 nm	1560 nm–1620 nm
		0.6π, −0.10 ps	0.3π, −0.05 ps	0.8π, −0.02 ps
